# Cerebrotendinous xanthomatosis: a literature review and case study

**DOI:** 10.3389/fcvm.2024.1496442

**Published:** 2024-12-09

**Authors:** Anthony Matta, Fabienne Ory Magne, Thierry Levade, Fabrice Bonneville, Jean Ferrières

**Affiliations:** ^1^ Department of Cardiology, Civilian Hospitals of Colmar, Colmar, France; ^2^ School of Medicine and Medical Sciences, Holy Spirit University of Kaslik, Jounieh, Lebanon; ^3^ INSERM, UMR1214 Toulouse NeuroImaging Centre “TONIC,” Centre Expert Parkinson de Toulouse, Service de Neurologie, CHU de Toulouse, Toulouse, France; ^4^ Institut National de la Santé et de la Recherche Médicale (INSERM) UMR1037, Centre de Recherches en Cancérologie de Toulouse (CRCT), Université Paul Sabatier, Laboratoire de Biochimie, Institut Fédératif de Biologie, CHU Purpan, Toulouse, France; ^5^ Department of Neuroradiology, Toulouse University Hospital, Toulouse, France; ^6^ Department of Cardiology, INSERM UMR 1295, Toulouse University Hospital, Toulouse, France

**Keywords:** cerebrotendinous xanthomatosis, cholestanol, bile acid synthesis, chenodeoxycholic acid, review

## Abstract

Cerebrotendinous xanthomatosis (CTX) is a rare but treatable inherited neurometabolic disorder that can lead to severe sequelae if left untreated. Chenodeoxycholic acid is a safe and effective treatment for CTX. Early diagnosis is essential to improve patient outcomes. Neurological disturbances, cataracts, and intractable diarrhea are key features to raise diagnostic suspicion and differentiate CTX from other metabolic disorders in patients with dyslipidemia and xanthomas. The diagnosis of CTX depends on high cholestanol plasma levels, undetectable plasma bile acids, neuroradiological findings, and CYP27A1 gene analysis. This review provides a stepwise approach to diagnosing patients with CTX, aims to improve physician awareness of CTX, and highlights the effectiveness of chenodeoxycholic acid as the standard of care. In addition, we report a unique case of CTX with major premature cardiovascular events, initially misdiagnosed as heterozygous familial hypercholesterolemia. This review also provides evidence to establish the c.470T>C (p. Leu157Pro) variant of the CYP27A1 gene as a likely pathologic variant.

## 1 Introduction

Cerebrotendinous xanthomatosis (CTX), first described in 1937, is a rare and often underdiagnosed autosomal recessive lipid storage disease caused by biallelic pathogenic variants of the CYP27A1 gene encoding sterol 27-hydroxylase (1). This inherited lipid disorder affects hundreds of patients worldwide (1). Its pathophysiology stems from a deficiency of this enzyme, which prevents cholesterol conversion into bile acids, thereby reducing intestinal fat absorption and leading to the accumulation of cholesterol and cholestanol (a saturated cholesterol derivative) in various organs, especially the central nervous system, tendons, lens, and arteries. If left untreated, CTX leads to serious neurological sequelae. The gold-standard treatment is chenodeoxycholic acid, which has proven its efficacy and safety in managing CTX. The clinical presentation of CTX varies with age, including infantile-onset diarrhea, childhood-onset cataracts, adolescent- to young adult-onset tendon xanthomas, and adult-onset progressive neurologic impairment (1). Unlike heterozygous familial hypercholesterolemia (HeFH), CTX patients present with highly variable and non-specific neurologic signs. The diversity of symptoms and non-specific findings usually contribute to significant diagnostic delays (2). This review reports a rare case of CTX in a adult French woman who presented with premature and predominant cardiovascular involvement, initially misdiagnosed as HeFH, and provides a short discussion of the pathophysiology, clinical manifestations, diagnosis, and management of CTX.

## 2 Case report

**Figure 1:**
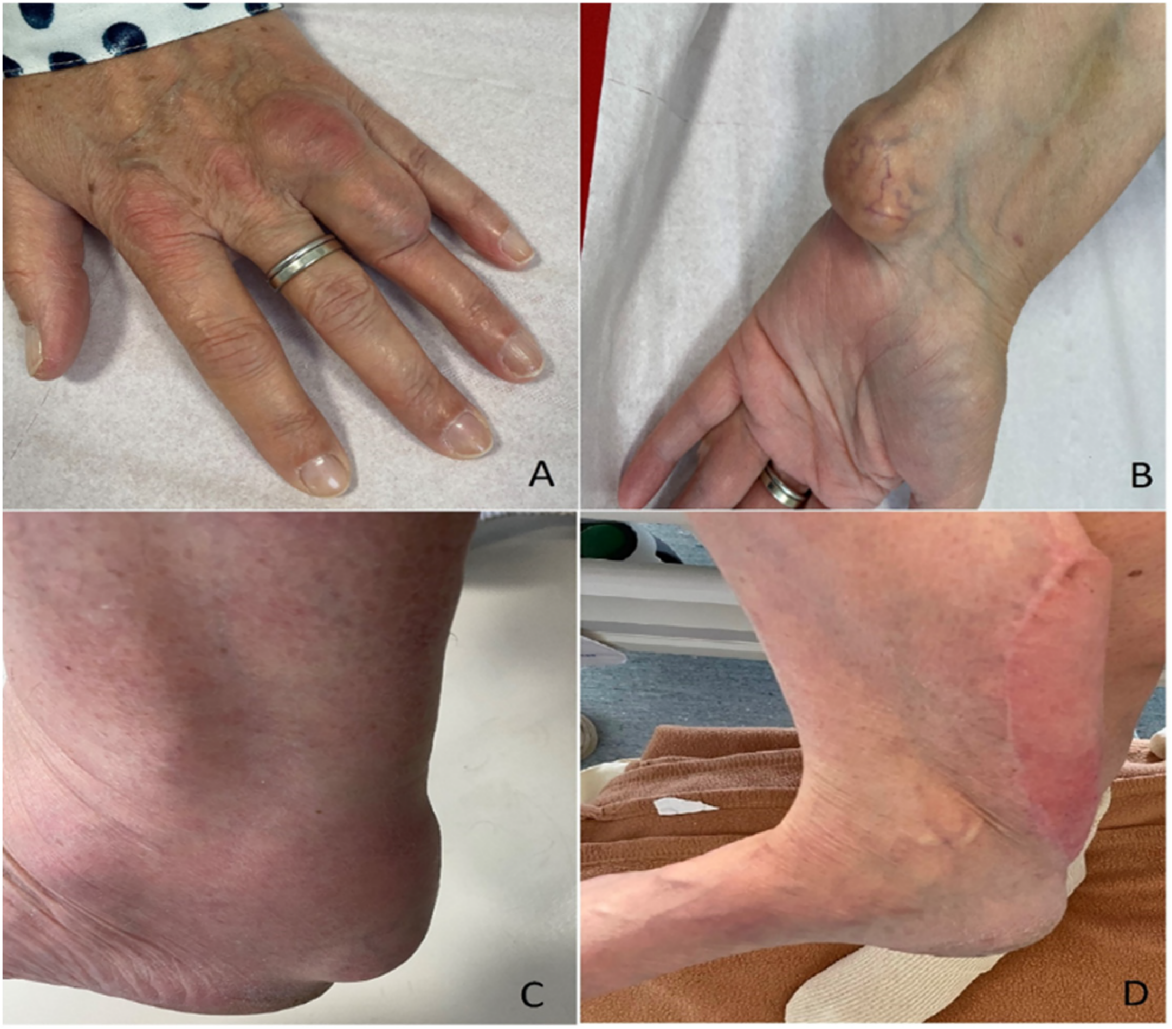
Xanthomas of the left extensor tendon of the hand **(A)**, left flexor tendon of the wrist **(B)**, and bilateral Achilles tendons **(C,D)**.

**Figure 2:**
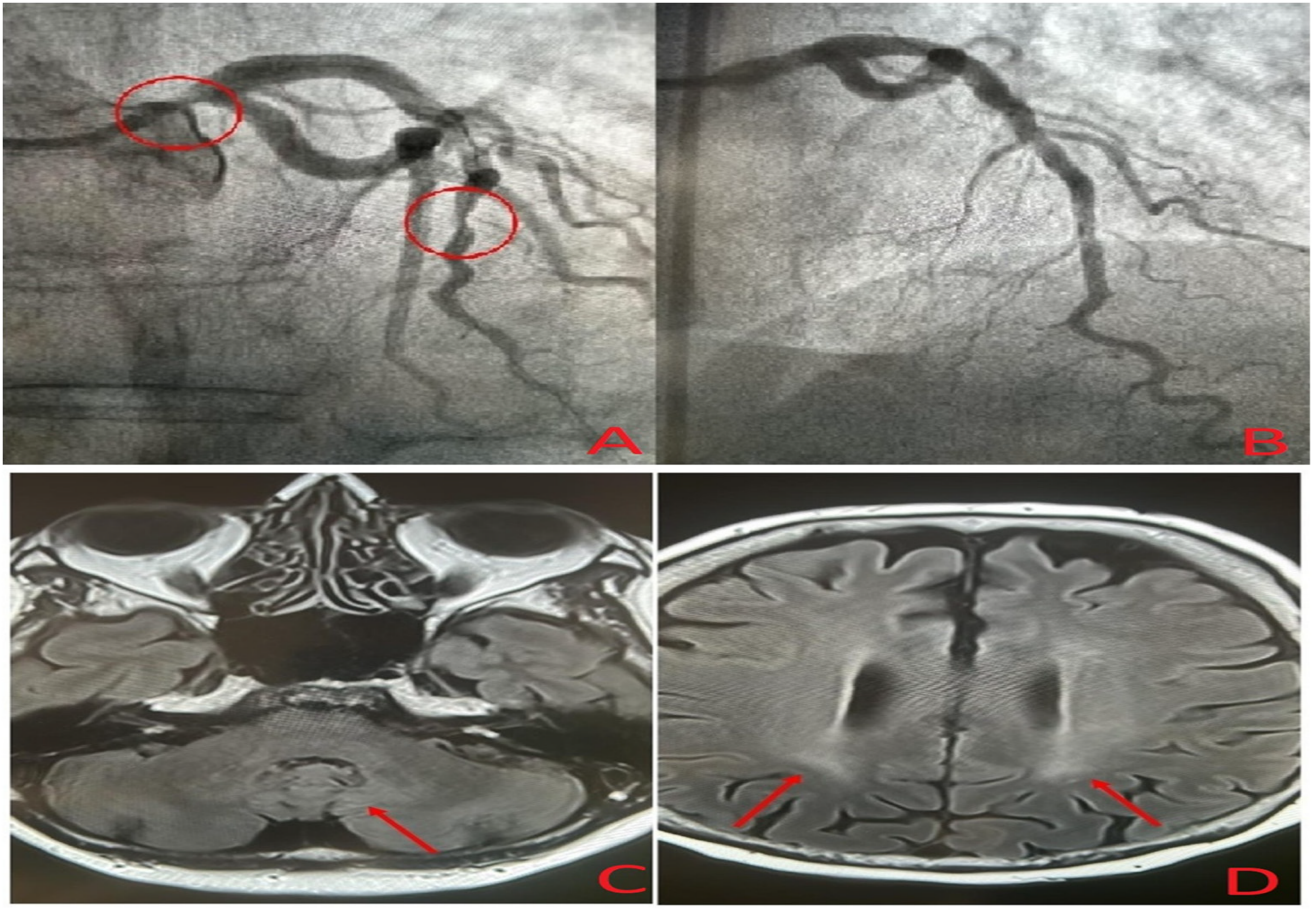
Coronary angiogram showing severe narrowing of the left main and left anterior descending coronary arteries **(A)**, post-treatment coronary angiogram after percutaneous coronary intervention **(B)**, and brain magnetic resonance imaging showing mild symmetrical FLAIR hyperintensities in the dentate nuclei **(C)** and periventricular white matter **(D)**.

**Figure 3:**
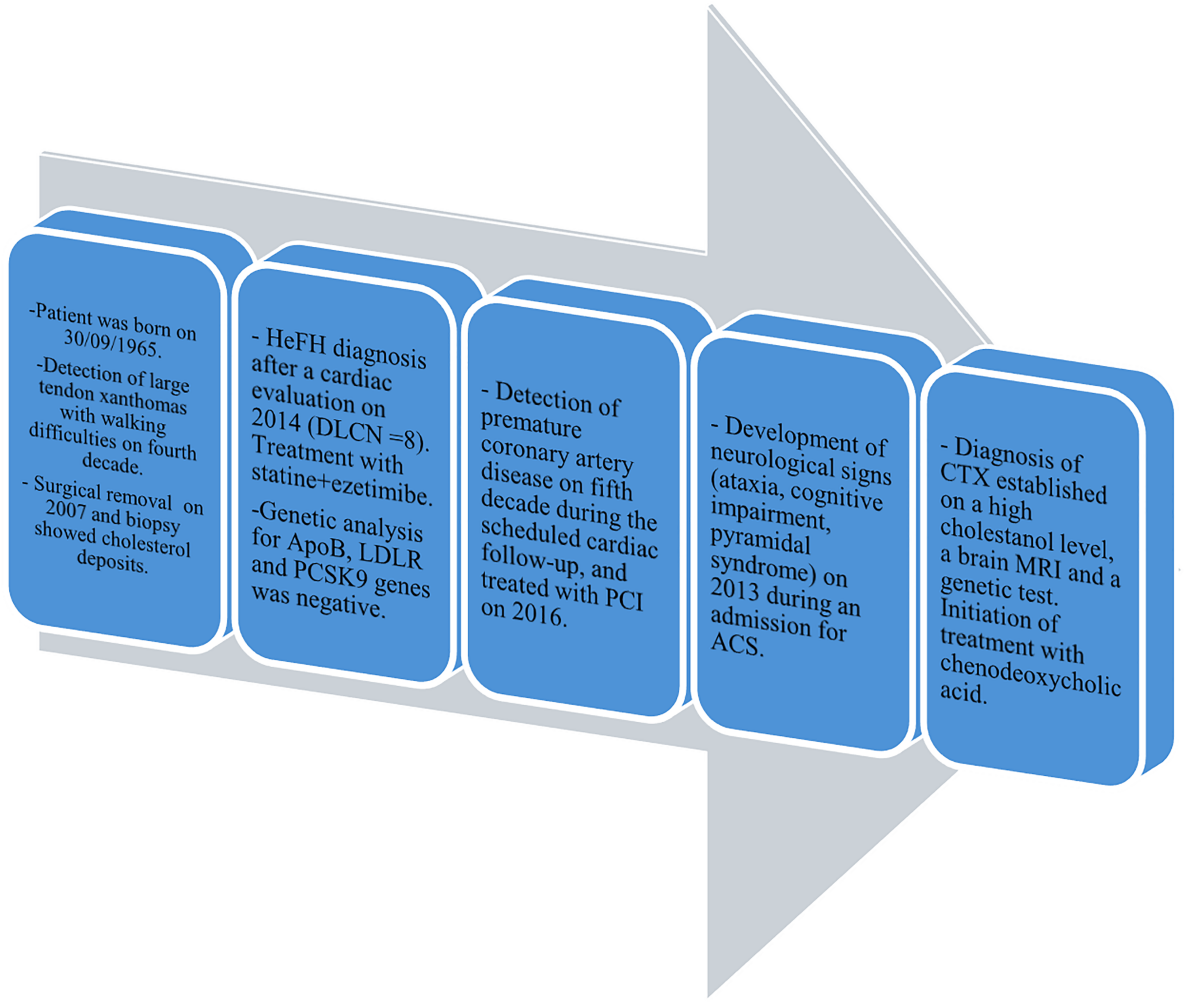
Timeline of the reported case of CTX. HeFH, heterozygous familial hypercholesterolemia; DLCN score, Dutch Lipid Clinic Network score; PCI, percutaneous coronary intervention; ACS, acute coronary syndrome.

A 49-year-old woman who was previously healthy presented to the Department of Cardiology at Toulouse University Hospital for a cardiac evaluation. Her medical history included surgical excision of a large right Achilles tendon xanthoma for cosmetic reasons and walking difficulties. Histopathologic examination of the specimen revealed cholesterol crystals. On admission, she was asymptomatic with normal vital signs and without additional cardiovascular risk factors. Her physical examination revealed xanthelasma, bilateral xanthomas of the Achilles tendon and flexor tendons of the wrist, and xanthomas of the left extensor tendons of the hand (Figure 1). Blood tests revealed a total cholesterol level of 234 mg/dl (6.05 mmol/L), LDL-cholesterol level of 141 mg/dl (3.65 mmol/L), HDL-cholesterol level of 50 mg/dl (1.29 mmol/L), and triglyceride level of 188 mg/dl (2.12 mmol/L). Genetic testing for genes encoding for the LDL receptor, PCSK9, apolipoprotein B, and apolipoprotein E came back negative. Her coronary artery calcium score was 0 Agatston units. Her Dutch Lipid Clinic Network score was 8. The patient was diagnosed with HeFH and treated with a combination therapy of ezetimibe/simvastatin (10/20 mg/day). Two years later, during a scheduled follow-up, she underwent coronary angiography based on the clinical judgment of the cardiologist, despite an assessed coronary calcium artery score of 6 Agatston units on non-contrast cardiac CT and an LDL-cholesterol level of 77 mg/dl (1.99 mmol/L). Coronary angiography showed severe narrowing of the left main and third segments of the left anterior descending coronary artery (LAD), both of which were treated with percutaneous coronary intervention and stent implantation (Figure 2). The patient was lost to follow-up during the COVID-19 pandemic and was admitted 7 years later for myocardial scintigraphy-induced acute anterior ST-segment elevation myocardial infarction. After percutaneous revascularization of a severe LAD narrowing on the coronary angiogram, the transthoracic echocardiography reported a left ventricular ejection fraction of 34%. During the index hospital stay, her physical examination revealed mild cerebellar ataxia (unable to walk in a straight line with a broad-based gait) and a pyramidal syndrome (brisk reflexes and bilateral Babinski sign). The investigations were completed with a neuropsychological assessment, which showed a mild cognitive impairment with a dysexecutive syndrome (mainly attention deficit). Brain magnetic resonance imaging revealed mild symmetrical fluid attenuated inversion recovery (FLAIR) hyperintensities in the periventricular white matter and dentate nuclei (Figure 2). The diagnostic workup was advanced by quantifying plasma sterols, followed by sequencing the CYP27A1 gene. An elevated level of cholestanol (46 μmol/L; upper control limit 16 μmol/L) was found, and two likely pathogenic variants in exons 3 (c.470T>C, p. Leu157Pro) and 6 (c.1184G>A, p. Arg395His) of CYP27A1 were identified. Analysis of CYP27A1 transcripts in peripheral blood leukocytes showed the presence of at least three types of transcripts. The first transcript carried the c.470T>C (r.470u>c) variation. The second transcript contained the 1184 substitution (r.1184g>a) at the very end of exon 6 and the entire intron 6. This transcript would result in a truncated protein because of a frameshift and a premature stop codon (p. Arg395GlnfsTer2). The third transcript lacked the last (3′-terminal) 89 nucleotides of exon 6, leading to the loss of residues Val366 to Arg395 and resulting in a truncated protein due to a premature stop codon at residue 412. To confirm that the variant was likely in trans, segregation analysis was performed. The patient's mother eventually agreed to genetic testing and was found to be heterozygous for the c.1184G>A variant. Quantification of bile acids by LC–MS/MS showed undetectable levels of primary bile acids, while 7α-hydroxy-4-cholesten-3-one, a bile acid precursor and diagnostic marker of CTX, was found to be elevated (488 nmol/L vs. 25–100 nmol/L in controls). Consequently, the patient was diagnosed with CTX and discharged on chenodeoxycholic acid at a dose of 250 mg three times daily (Figure 3). After 1 year of treatment, the levels of primary bile acids and 7α-hydroxy-4-cholesten-3-one (39 nmol/L) normalized.

## 3 Pathophysiology

**Figure 4:**
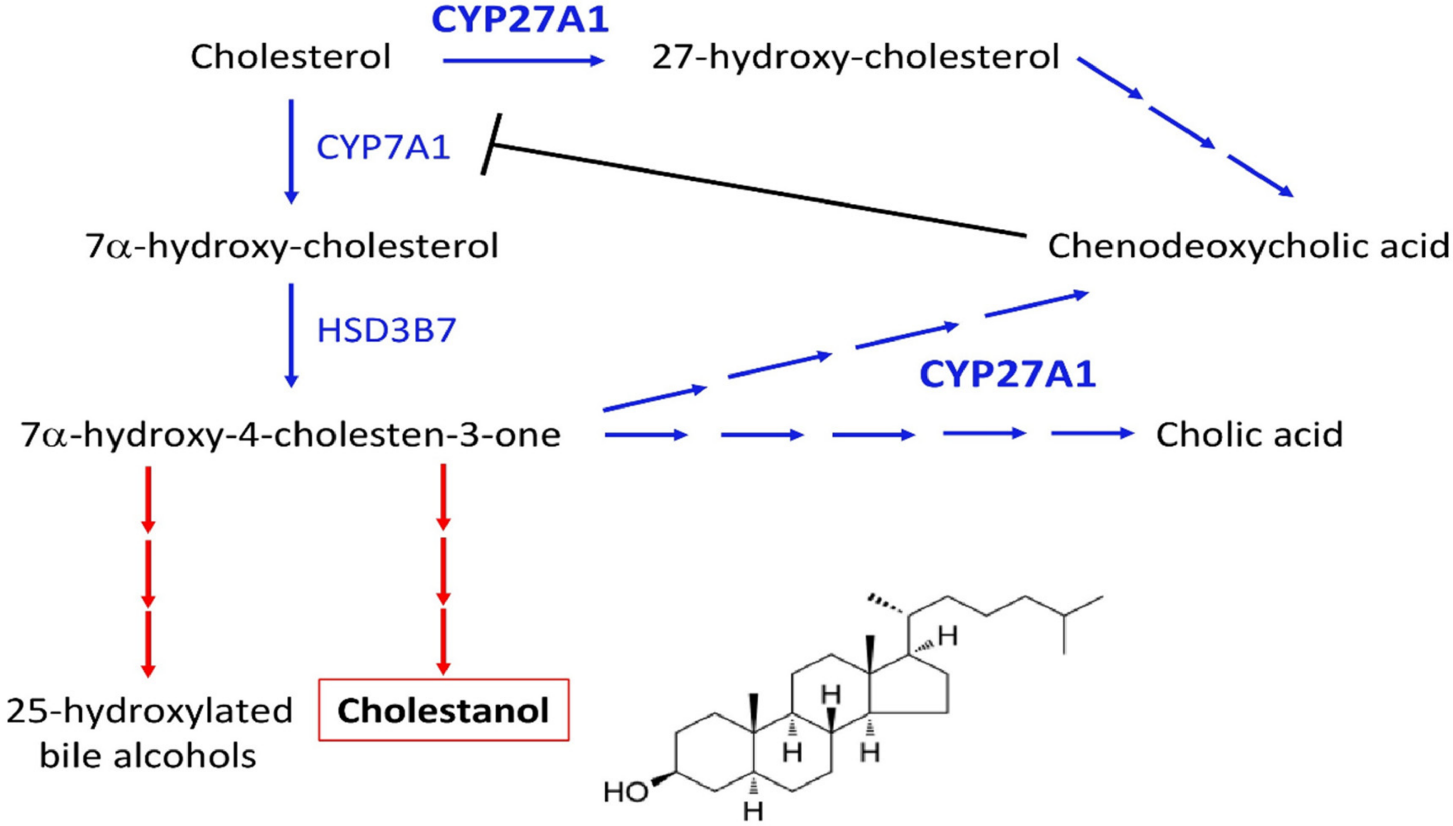
Synthesis pathway of cholestanol and the site of action of chenodeoxycholic acid.

**Figure 5:**
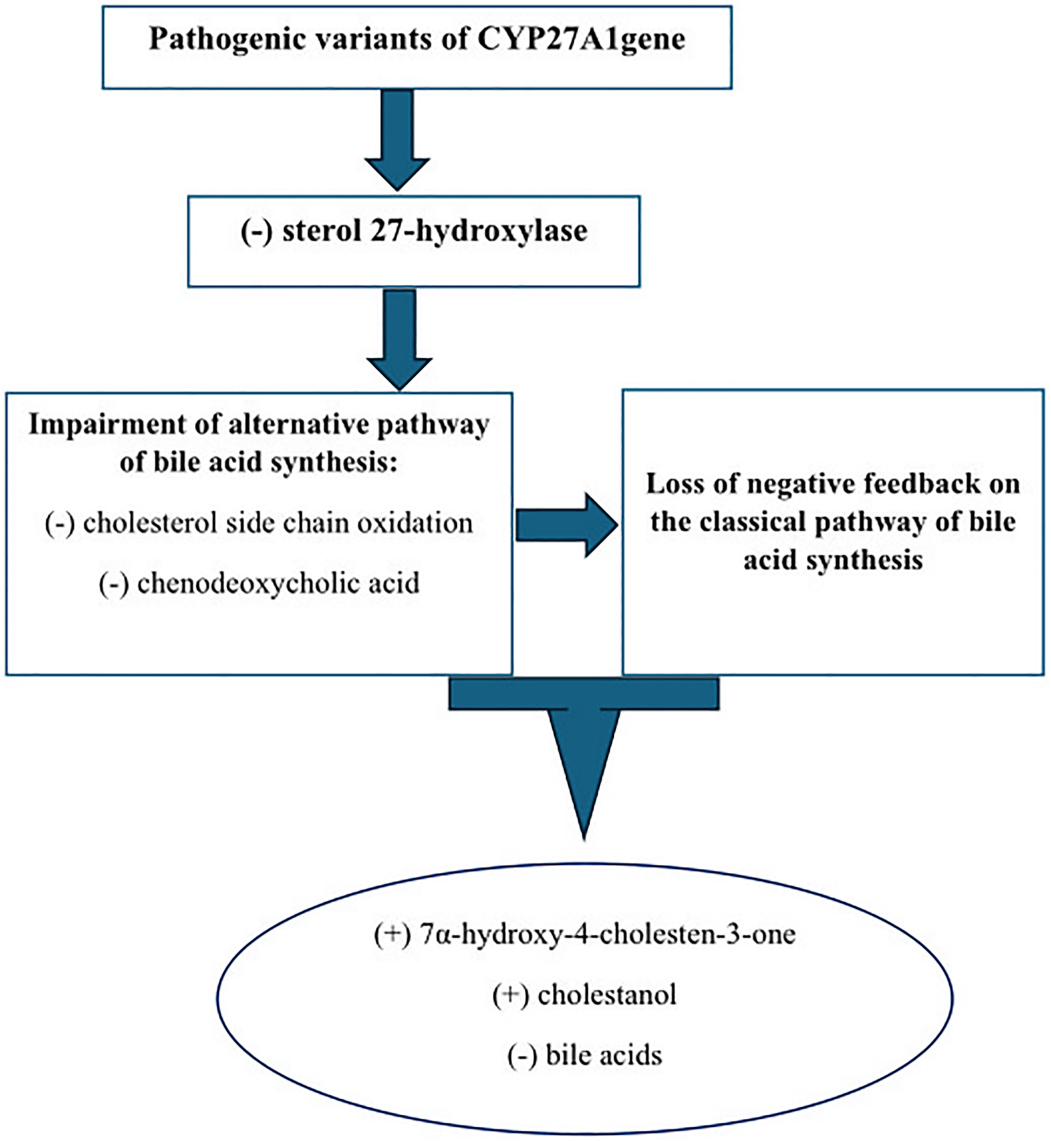
Pathophysiology of CTX.

Bile acids (cholic acid and chenodeoxycholic acid) are synthesized in the liver from cholesterol via two distinct pathways implicating the enzyme sterol 27-hydroxylase (CYP27A1). This process is negatively regulated by chenodeoxycholic acid, which suppresses the transcription of cholesterol 7α-hydroxylase (CYP7A1), the rate-limiting enzyme. Deficient activity of CYP27A1 results in reduced production of bile acids and abnormal production of cholestanol (also named dihydrocholesterol; InChIKey: QYIXCDOBOSTCEI-QCYZZNICSA-N) and 25-hydroxylated bile alcohols (Figure 4). The pathophysiology of CTX is based on the reduced activity of the mitochondrial enzyme sterol 27-hydroxylase, which plays a key role in cholesterol metabolism and bile acid synthesis pathways (3–6). This deficiency results from pathogenic variants of the CYP27A1 gene and impairs the alternative metabolic pathway of bile acid synthesis, compromising cholesterol side-chain oxidation and chenodeoxycholic acid formation (3–6). Then, the main consequences are the loss of negative feedback regulation by chenodeoxycholic acid on the classic pathway of bile acid synthesis, the upregulation of 7α-hydroxylase, and increased levels of 7α-hydroxy-4-cholesten-3-one and its metabolites, especially cholestanol (3–6) (Figure 5). The clinical manifestations and development of CTX result from cholesterol and cholestanol accumulation in various organs. The accumulated cholestanol mainly results from the conversion of 7α-hydroxy-4-cholesten-3-one into cholestanol and bile alcohols. To date, the deposition of cholestanol in the brain is not entirely understood, and three hypotheses have been proposed: increased blood–brain barrier permeability, cholestanol production in the brain from cholesterol and its intermediates, or conversion of 7α-hydroxy-4-cholesten-3-one to cholestanol by microglia, neurons, and astrocytes after crossing the blood–brain barrier (7). Pathological findings in CTX patients include numerous yellowish deposits in the Choroid plexus and brain white matter on macroscopic examination and various scattered lipid crystal clefts in the cerebellar hemispheres on microscopic examination (8).

## 4 Clinical overview of CTX

The clinical presentation of CTX is heterogeneous and highly variable, encompassing a wide spectrum of neurological and non-neurological signs and symptoms (Table 1). Cataracts, xanthomas, and progressive neurological deterioration represent the classic clinical manifestations. The disease progression is marked by four main clinical features: infantile-onset diarrhea, childhood-onset cataracts, adolescent- to young adult-onset tendon xanthomas, and adult-onset progressive neurologic function (1). CTX affects multiple systems, including the muscular, ocular, cardiovascular, enterohepatic, pulmonary, skeletal, and central and peripheral nervous systems (3). While chronic diarrhea is often the earliest clinical manifestation, cataracts are the first finding in 75% of CTX patients (1). The reported prevalence of principal clinical features is 40% for infantile-onset diarrhea, 89% for childhood-onset cataract, 78% for adolescent- to young adult-onset tendon xanthomas, 25% for cardiovascular findings, 67% for osteopenia, 60% for intellectual disability, 44% for psychiatric disturbances, 36% for ataxia, 64% for spastic paraparesis, 9% for parkinsonism, 70% for peripheral neuropathy, and 33% for seizures (9). The pyramidal and/or cerebellar signs secondary to the involvement of the cerebellum cortex, corticospinal tracts, subcortical white matter, and dentate nuclei are almost invariably present (10, 11). The extrapyramidal neurological signs are late clinical manifestations of CTX (1). Until now, no genotype–phenotype correlations have been established for CYP27A1 (1).

**Table 1:** Summary of the main symptoms of cerebrotendinous xanthomatosis (F, female; M, male).

Symptoms	Common time of onset	Prevalence	Reported in this case
Infantile-onset diarrhea	Infancy	40%	No
Cataract	Adulthood (first decade)	89%	No
Palpebral xanthelasmas	Peak in the fourth–fifth decade	32% (F), 17% (M)	Yes
Tendon xanthomas	Second or third decade	78%	Yes
Premature atherosclerosis and coronary artery disease	Fourth and fifth decades	25%	Yes
Osteoporosis	Adulthood	67%	No
Intellectual disability	Third decade	60%	Yes
Pyramidal and/or cerebellar signs	Second and third decades	64%	Yes
Extrapyramidal signs	Late stage of disease		
Ataxia		36%	Yes
Parkinson’s disease		9%	No
Seizures	Adulthood	33%	No
Peripheral neuropathy	Adulthood	70%	No
Neuropsychiatric disturbances	Adulthood	44%	No
Cholestasis, gallstones, hepatitis	Infancy	Rare	No

## 5 Diagnosis workup

**Figure 6:**
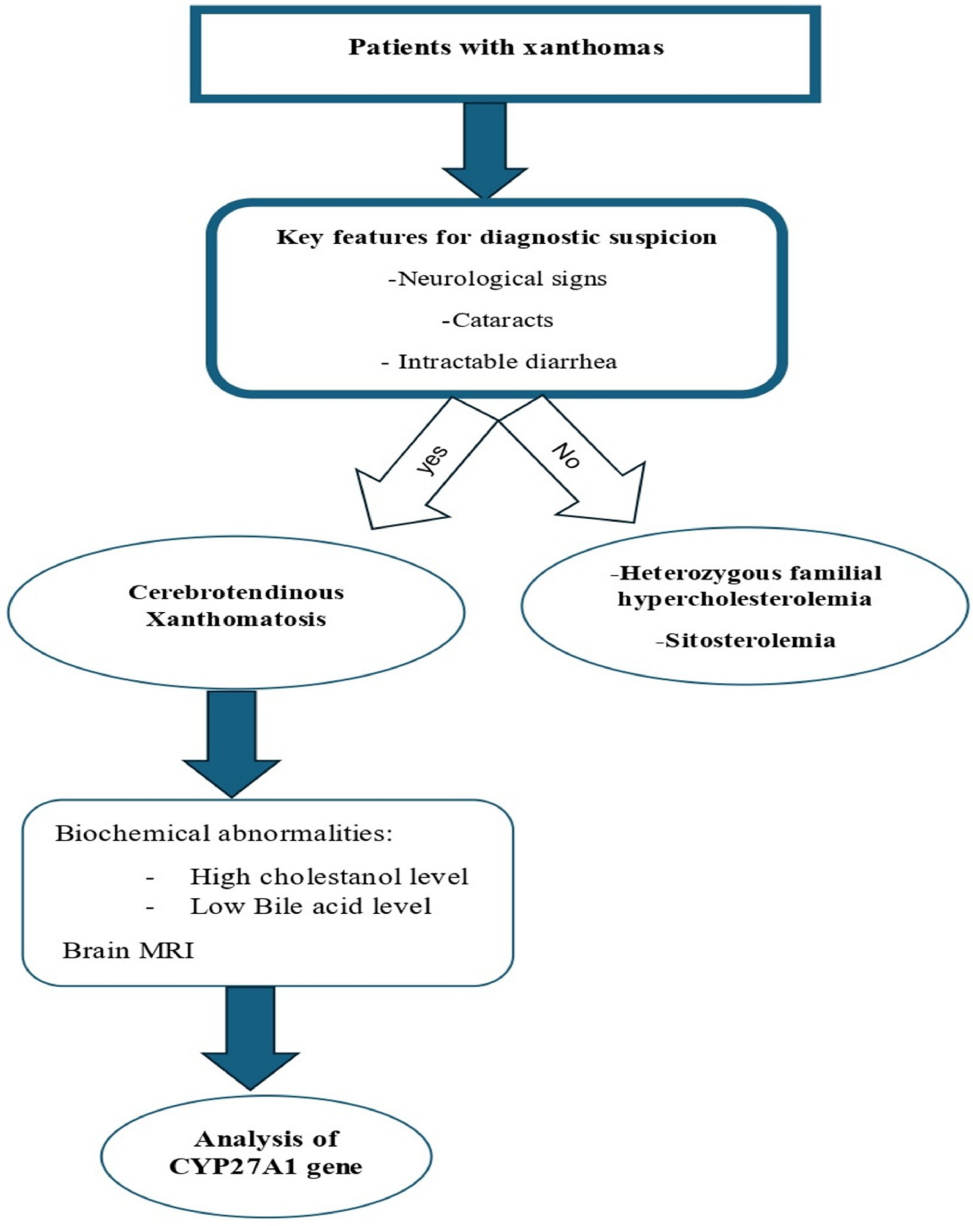
Stepwise diagnostic approach for patients with xanthomas.

**Figure 7:**
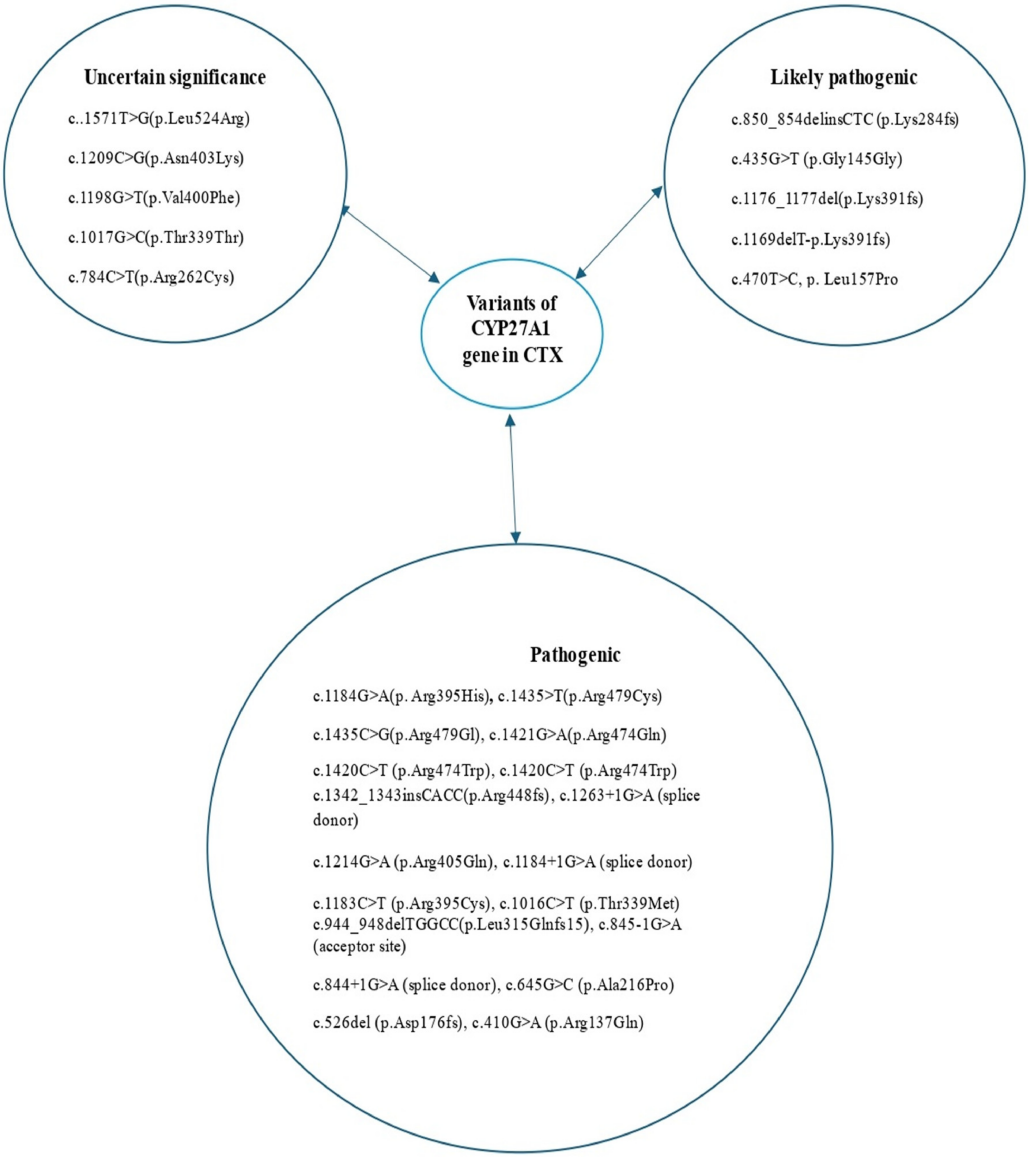
Variants of the CYP27A1 gene reported in patients with cerebrotendinous xanthomatosis.

An average delay of 16 years between symptom onset and time of diagnosis has been reported in CTX (12). The four pillars of CTX diagnosis are clinical, biochemical, neuroradiological, and genetic findings (Figure 6). A high cholestanol plasma level is the hallmark biomarker of CTX (5). The biochemical abnormalities found in CTX patients include elevated concentrations of other cholesterol precursors like 7- and 8-dehydrocholesterol (13), high levels of bile alcohols such as glucuronides in plasma and urine, increased bile acid precursors in plasma and bile, and decreased chenodeoxycholic acid levels. It is noteworthy that a few cases of atypical CTX with normal or near-normal cholestanol levels have been reported; however, in these cases, elevated levels of bile acid precursors and bile alcohols were found (14). The diagnosis was genetically and clinically confirmed by the presence of large extensor tendon xanthomas (14). Therefore, a normal plasma cholestanol level is insufficient to exclude a diagnosis of CTX. Brain MRI plays a significant role in the diagnosis, especially in CTX patients with neurological signs. The most distinctive findings of CTX include increased signal intensity on T2-weighted and/or FLAIR images in the dentate nuclei and the surrounding cerebellar white matter(2). Different neuroradiological findings depending on the clinical status of the patient could also be detected. Recently, cerebellar vacuolization on T1-weighted and/or FLAIR images has been identified as a predictor of poor prognosis (2). The gold-standard test for a definitive diagnosis of CTX is the identification of biallelic pathogenic CYP27A1 variants. To date, several pathogenic and likely pathogenic variants have been identified, including c.1435>T (p.Arg479Cys), c.1435C>G (p.Arg479Gly), c.1421G>A (p.Arg474Gln), c.1420C>T (p.Arg474Trp), c.1342_1343insCACC (p.Arg448fs), c.1263+1G>A (splice donor), c.1214G>A (p.Arg405Gln), c.1184+1G>A (splice donor), c.1183C>T (p.Arg395Cys), c.1176_1177del (p.Glu392fs), c.1169delT (p.Lys391fs), c.1016C>T (p.Thr339Met), c.944_948delTGGCC (p.Leu315Glnfs15), c.850_854delinsCTC (p.Lys284fs), c.845-1G>A (acceptor site), c.844+1G>A (splice donor), c.645G>C (p.Ala216Pro), c.526del (p.Asp176fs), c.435G>T (p.Gly145Gly), and c.410G>A (p.Arg137Gln) (7) (Figure 7). The c.1184G>A variant has previously been described in a homoallelic patient with CTX. However, the c.470T>C variant is very rare, has not yet been published in the literature, and is still classified as a variant of uncertain significance. The American College of Medical Genetics and Genomics (ACMG) criteria given by GeneBe and hg38 InterVar are PM2 PP3 and PM1 PM2, respectively. For instance, prior to our report, according to GeneBe, it is stated that “The NM_000784.4 (CYP27A1): c.470T>C (p. Leu157Pro) variant causes a missense change involving the alteration of a non-conserved nucleotide. The variant allele was found at a frequency of 0.000000684 in 1,461,838 control chromosomes in the GnomAD database, with no homozygous occurrence. In-silico tool predicts a pathogenic outcome for this variant. Variant has been reported in ClinVar as Uncertain significance (VUS).” The addition of the following two criteria, PM3 (recessive disorders, detected in trans with a pathogenic variant) and PP4 (patient phenotype or family history is highly specific for a disease with a single genetic etiology), thus reclassifies the c.470T>C variant from class 3 (VUS) to class 4 or likely pathogenic.

## 6 Differential diagnosis and management

CTX mimics sitosterolemia and hypercholesterolemia, both of which can also manifest with tendon xanthomas. However, the absence of neurological symptoms, cataract formation, and intractable diarrhea help to differentiate CTX from sitosterolemia and hypercholesterolemia. Also, these classic manifestations distinguish CTX from Smith–Lemli–Optiz syndrome, other inborn disorders of bile acid metabolism, and non-specific liver diseases that share some clinical features with CTX (15–17). Hypercholesterolemia is also characterized by elevated cholesterol levels but with normal cholestanol levels and a family history of premature atherosclerosis. However, CTX patients typically exhibit elevated cholestanol levels but normal or low cholesterol levels (18). In the present atypical case with an unusual disease course, the patient was initially misdiagnosed with HeFH, most likely due to the lack of neurologic signs, the existence of premature cardiovascular events, and moderately elevated LDL-cholesterol levels. We retrospectively understood that cerebral ataxia may have been probably masked during the first clinical evaluation due to the presence of large tendon xanthomas, which contributed to walking abnormalities. In real-world settings and in line with this case report, a delayed diagnosis of CTX is often observed (18). One should emphasize the importance of a detailed clinical history and careful examination of patients with lipid disorders, especially at an early age. For example, the presence of developmental delay and learning difficulties during childhood and/or cognitive decline in adulthood points toward CTX (2). Supplementation with chenodeoxycholic acid, the standard of care for CTX patients, inhibits bile acid synthesis by negative feedback on the classic pathway, drastically decreasing cholestanol production and its accumulation in tissues (19–21). Improvement or stabilization of systemic and neurological manifestations, including cognitive impairment, pyramidal and cerebellar signs, and peripheral neuropathy has been observed (2). Thus, early diagnosis and treatment are crucial to improving the life expectancy and prognosis of patients with CTX. It is noteworthy that monotherapy with chenodeoxycholic acid is recommended as first-line treatment (22). There is no additional benefit for combined therapy with HMG-CoA (hydroxymethylglutaryl-CoA) reductase (statins) and/or LDL apheresis (22).

## 7 Conclusion

CTX is an uncommon but treatable metabolic disorder, making diagnosis at an early age extremely important. The heterogeneity of symptoms poses a significant challenge facing physicians; thereby, searching for neurological signs, cataracts, and intractable diarrhea may help differentiate CTX from HeFH, a common differential diagnosis. This review helps improve physicians’ awareness of CTX for early diagnosis and treatment. We emphasize the importance of paying careful attention to physical examinations, clinical manifestations, and medical history of patients with lipid disorders. It is extremely important to spread awareness among healthcare professionals about this rare disorder to ensure appropriate treatment before disease progression. This care reflects the importance of teamwork (internal medicine, neurologist, and cardiologist) in the management of patients, especially those with uncommon diseases.
